# The prevalence of mental distress before the Great East Japan Earthquake and the associated impact of an aged society: An ecological study

**DOI:** 10.1371/journal.pone.0203985

**Published:** 2018-09-26

**Authors:** Jimpei Misawa, Rie Ichikawa, Akiko Shibuya, Yukihiro Maeda, Teruyoshi Hishiki, Yoshiaki Kondo

**Affiliations:** 1 Department of Health Care Services Management, Nihon University School of Medicine, Itabashi, Tokyo, Japan; 2 Department of Pediatrics and Child Health, Nihon University School of Medicine, Itabashi, Tokyo, Japan; 3 Department of Information Science, Faculty of Science, Toho University, Funabashi, Chiba, Japan; Hamamatsu Ika Daigaku, JAPAN

## Abstract

Various studies have determined that the Great East Japan Earthquake (GEJE) caused mental distress among residents in affected areas. However, previous studies had not considered the prevalence of mental distress before the GEJE, and ignored the impact of an aged society on mental distress. Therefore, we aimed to describe the prevalence of mental distress before the GEJE in Miyagi Prefecture, Japan and elucidate the effect of an aged society on mental distress. We conducted an ecological study, using municipality in Miyagi Prefecture as the study unit. We used the cross-sectional mail survey data conducted in February 2011. We performed a correlation analysis in each of the 39 municipalities in Miyagi Prefecture. The prevalence of serious mental distress was 9.1%. The proportion of the population aged 65 years or older was related to the prevalence of serious mental distress in municipalities with a low proportion of all workers engaged in primary industry and with a high estimated number of inpatients with mental illness. We found that residents in Miyagi Prefecture suffered from poor mental health before the GEJE. Aged society was related to serious mental distress in the areas with advanced industrial structure and more patients with mental illness. We should approach mental health problems in the context of social structure, particularly in an aged society, based on facts about mental distress before the GEJE.

## Introduction

**Fig 1:**
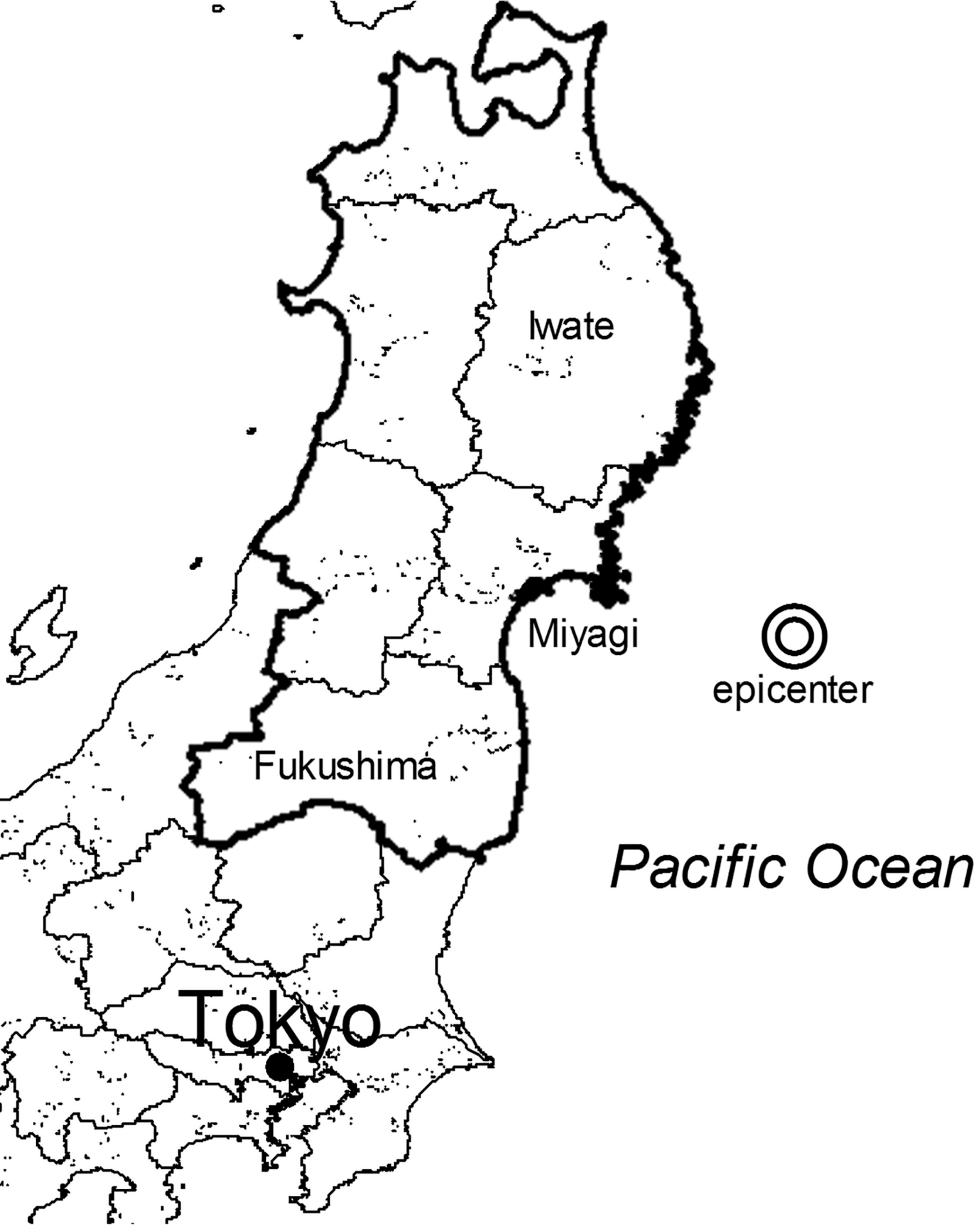
Map showing the epicenter of the Great East Japan Earthquake. The epicenter of the Great East Japan Earthquake is located on the Pacific Ocean, about 130 km away from Miyagi Prefecture. The bold boundary line represents Tohoku region. The figure was edited and processed by the authors using the National Land Numerical Information (Administrative Zones Data) by Policy Bureau, Ministry of Land, Infrastructure, Transport and Tourism.

The Great East Japan Earthquake (GEJE) occurred on March 11, 2011 on the coast of northeast Japan, resulting in the death or disappearance of approximately 20,000 people [1]. The GEJE mainly affected the Iwate, Miyagi, and Fukushima Prefectures, in the Tohoku region of Japan (Fig 1). Most of the damage to the houses or harm to humans was however, concentrated in Miyagi Prefecture. The earthquake not only caused damage or harm to houses and humans, but it also caused damage to the mental health of the residents of this Prefecture. A substantial proportion of the affected individuals experienced psychological distress as a result [2]. According to surveys conducted in Miyagi Prefecture, between 5–8% of the population [3–6], 3.5–4.4% of the public servants [7,8], and 10–40% of the refugees in their temporary houses [9,10] suffered from mental distress.

However, previous studies did not consider the prevalence of mental distress before the GEJE. Moreover, since they examined only specific municipalities or participants who had suffered great damage/harm, it may have been difficult to clearly determine the distribution of existing mental health problems in the whole area. It is unlikely that it would be meaningful to discuss the distribution of mental distress after the GEJE without clarifying the distribution of mental distress before the GEJE. However, previous studies have not shown the distribution of the prevalence of mental distress before the GEJE. Thus, it is necessary to provide a clear description of the prevalence of mental distress before the GEJE in the whole area. By describing the prevalence of mental distress before the GEJE, we are able to provide fundamental data that is useful to compare conditions of mental distress before and after the GEJE.

Furthermore, if we can not only identify the distribution of the prevalence of mental distress before the GEJE, but also identify relevant essential social factors related to the prevalence of mental distress before the GEJE, we can propose suggestions for preventive health policies to assist in the substantial improvement of mental health problems post-disaster. Previous studies have shown that social factors at mezzo or individual levels, such as social support of relations [3,10–14], social participation [15], and community characteristics including social capital [6,9], assisted in alleviating serious mental distress after the GEJE. As a result of these findings, the importance of continual mental health interventions for residents of disaster areas has been highlighted in Japan. However, the purpose of modern public mental health policies has been to improve psychosocial health by addressing factors of mental health in all public policy spheres [16]. Thus, it would be necessary to address mental health problems from a social structural view. However, previous studies have ignored the effect of social structure as a construct at an ecological or macro level in contemporary Japanese society. Since psychosocial status is affected by social structure [17], it is necessary to clarify relevant factors from a social structural view to address mental distress. In studies that only discuss social factors at mezzo or individual levels, it is difficult to identify policy solutions for mental health distress. Thus, we need to discuss mental distress from a social structural viewpoint in contemporary Japanese society.

The most serious social structural problem in contemporary Japan is an aged society, as the proportion of elderly people over 65 years old is high. Previous studies have shown that an aged society was associated with economic problems of unemployment [18,19], and that economic problems (i.e. unemployment) were related to the deterioration of mental health [11,20,21]. Thus, we hypothesize that an aged society could be associated with mental distress. However, to our knowledge, studies have not investigated the relationship between an aged society and mental distress. As we predict that the proportion of elderly people over 65 years old will increase in the future, it is important to consider the relationship between an aged society and mental health.

Therefore, the aims of this study were to provide a description of the prevalence of mental distress before the GEJE in Miyagi Prefecture, Japan and to elucidate the effect of an aged society on mental distress before the GEJE. Findings from this study will provide fundamental data useful for comparing mental distress before and after the GEJE, and will assist in the identification of policy solutions for mental health distress from a structural viewpoint.

## Methods

### Study design

**Fig 2:**
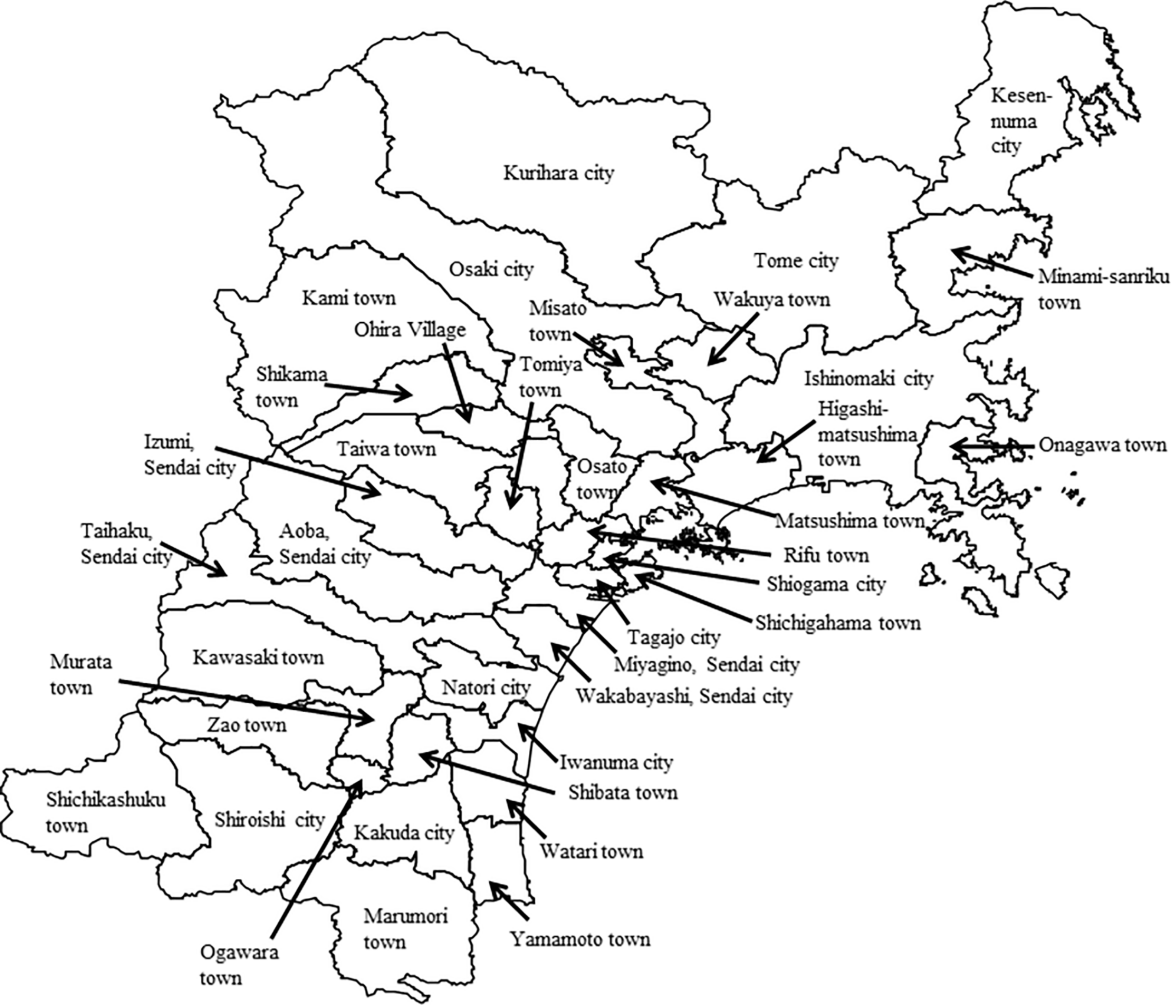
Map showing the municipalities in Miyagi Prefecture. Miyagi Prefecture is composed of five wards of Sendai City and 34 municipalities. Therefore, we analyzed 39 municipalities in total. The figure was edited and processed by the authors using the National Land Numerical Information (Administrative Zones Data) by Policy Bureau, Ministry of Land, Infrastructure, Transport and Tourism.

We conducted an ecological study, using municipalities in Miyagi Prefecture as the study unit (N = 39). Miyagi Prefecture is located approximately 300 km north of Tokyo, and consists of 39 municipalities (Fig 2). The population of Miyagi Prefecture is about 1.9 million.

### Data

#### Mental distress data by social survey

We used the cross-sectional mail survey data to determine the prevalence of mental distress. We conducted the survey in February 2011. Subjects were 2,500 randomly selected residents of Miyagi Prefecture, aged 20−74 years. With an estimation error of 2.5%, we needed to extract 1,500 samples to estimate the precise prevalence in the population of approximately 1.9 million. Therefore, assuming a response rate of 60%, we required 2,500 samples. We chose the sample according to the population size of each municipality. The sample size from each municipality was determined according to population size, with at least approximately 30 samples from each location (Table 1). The survey was self-administered by those willing to participate in the study. The survey received 1,543 responses (response rate: 62.5%). This study was approved by the Tohoku University Medical Sciences' ethical review board (No. 2010–245, September 15, 2010). We interpreted the voluntary return of the self-administered questionnaire in the postal survey as informed consent. This consent procedure was also approved by the ethical review board above. The data were analyzed anonymously. The data are available from a supporting information file (S1 Dataset).

**Table 1:** Sample size in each municipality.

Municipalities	Population *	Sample size	Respondents	Response rate (%)
Aoba, Sendai City	226,993	122	69	56.6
Miyagino, Sendai City	149,757	80	33	41.3
Wakabayashi, Sendai City	105,586	57	33	57.9
Taihaku, Sendai City	177,484	96	57	59.4
Izumi, Sendai City	170,093	92	57	62.0
Ishinomaki City	135,035	110	70	63.6
Shiogama City	48,291	68	43	63.2
Kesen-numa City	62,345	75	43	57.3
Shiroishi City	31,719	60	36	60.0
Natori City	57,543	73	37	50.7
Kakuda City	26,601	58	34	58.6
Tagajo City	50,217	69	36	52.2
Iwanuma City	35,406	62	37	59.7
Tome City	71,360	80	45	56.3
Kurihara City	65,093	77	48	62.3
Higashi-matsushima City	34,849	62	45	72.6
Osaki City	111,183	99	59	59.6
Zao Town	10,909	50	28	56.0
Shichikashuku Town	1,496	47	26	55.3
Ogawara Town	19,009	54	30	55.6
Murata Town	10,164	50	27	54.0
Shibata Town	31,549	60	34	56.7
Kawasaki Town	8,532	49	21	42.9
Marumori Town	13,540	52	36	69.2
Watari Town	29,126	59	43	72.9
Yamamoto Town	14,397	52	30	57.7
Matushima Town	13,202	51	31	60.8
Shichigahama Town	16,830	53	31	58.5
Rifu Town	26,607	58	38	65.5
Taiwa Town	19,906	55	35	63.6
Osato Town	7,694	49	34	69.4
Tomiya Town	35,634	62	38	61.3
Ohira Village	4,407	47	31	66.0
Shikama Town	6,260	48	29	60.4
Kami Town	21,895	56	37	66.1
Wakuya Town	15,014	52	33	63.5
Misato Town	21,485	55	31	56.4
Onagawa Town	8,658	49	31	63.3
Minami-sanriku Town	14,673	52	31	59.6
Unknown	0	0	2	-
Total	1,910,452	2,500	1,489	59.6

* Population aged at least 20 years and listed on the voter list, as of September 2010.

We used the Japanese version of the Kessler Psychological Distress Scale (K6) [22,23] to measure mental distress in the survey. The validity of the use of the Japanese K6 among the Japanese population has been confirmed as detailed in a previous publication [24]. The K6 is used to assess non-specific psychological distress. The Japanese version consists of six items which ask how frequently respondents have experienced the following symptoms during the past 30 days: “feeling so sad that nothing could cheer you up,” “feeling nervous,” “hopeless,” “restless or fidgety,” “feeling that everything was an effort,” and “feeling worthless.” Each item was scored according to a five-point scale; responses of “none of the time” were allocated a score of 0 while responses of “all of the time” were allocated a score of four. The sum of scores for the six items (ranging from 0 to 24) was used to indicate severe mental disorders [25], high scores indicated a poor mental health status. A score of 13 or more was defined as serious mental distress as per the international standards for the classification of serious mental distress [26]. Excluding the participants who did not respond to the questions regarding K6, we analyzed the remaining responses (n = 1,487, final response rate: 59.6%) and calculated the serious mental distress rate for each of the municipalities.

#### Social structural data

We determined the proportion of the population aged 65 years or older (continuous) to measure the aged society for each municipality. Additionally, we employed social structural characteristics from the following perspectives of urbanization, economic, industrial, and health determinants to examine the effect of the proportion of the population aged 65 years or older on the prevalence of serious mental distress. We used the population density of the residential area (number of people living per km^2^) as an urbanization determinant of social structure to measure the degree of urbanization for each municipality. The unemployment rate was used as an economic determinant of social structure to measure the magnitude of unemployment in each municipality. We used the proportion of all workers engaged in primary industry as an industrial determinant of social structure to measure the industrial structure in each municipality. Suicide rate (per 100,000 population) and the estimated number of inpatients with mental illness (per day) were used as health determinants of social structure, to measure suicide and mental illness in each municipality, respectively.

We used the national census data from Miyagi Prefecture conducted in 2010, which was published by the National Statistics Center [27], for information on the proportion of the population aged 65years or older, unemployment rate, and the proportion of all workers engaged in primary industry. We used data from “the statistical observations of Shi, Ku, Machi, Mura 2012,” published by Statistics Japan [28], for information regarding the population density of residential areas as of 2010. Statistics on population and residential area are required to calculate the population density of residential areas. Since these statistics of 2010 are listed in the dataset of “the statistical observations of Shi, Ku, Machi, Mura 2012,” we used this dataset. We used the national data on suicide of 2009 and 2010, which was published by the Ministry of Health, Labor and Welfare [29], and determined the average value of both years as the suicide rate, as the suicide rate fluctuates greatly even if the number of suicides is small in municipalities with little population. However, because the suicide counting method before 2008 was different from the current one, we adopted only two years, from 2009 to 2010. Patient survey data from 2008, published by the Ministry of Health, Labor and Welfare [30], was used to obtain the estimated number of inpatients with mental illness (per day). Patient surveys were conducted once every three years. We used the 2008 survey data as the 2011 data would have been influenced by the GEJE, and would not have provided an accurate representation of mental illness before the GEJE. The estimated number of inpatients with mental illness was calculated not in a municipal unit but in a secondary medical care area unit. The secondary medical care area is a regional zone, composed of several municipalities. Table 2 presents a brief summary of these social structure data. These data are available from a supporting information file (S2 Dataset).

**Table 2:** Summary of social structural data.

Social structural data		Source	Area unit	Year of analysis
An aged society				
	The proportion of the population aged 65 years or older	National census data 2010 [27]	Municipality	2010
Urbanization determinants				
	Population density of residential area	Statistical observations of Shi, Ku, Machi, Mura 2012 [28] ^a^	Municipality	2010
Economic determinants				
	Unemployment rate	National census data 2010 [27]	Municipality	2010
Industrial determinants				
	The proportion of all workers engaged in primary industry	National census data 2010 [27]	Municipality	2010
Health determinants				
	Suicide rate	National data on suicide [29]	Municipality	2009–2010 ^d^
	The estimated number of inpatients with mental illness	Patient survey 2008 [30] ^b^	Secondary medical care area ^c^	2008

a Information on the population and residential area as of 2010 are not included in the statistical observations of Shi, Ku, Machi, Mura 2010 and 2011 versions.

b Since patient surveys are conducted once every three years, we employed the 2008 data for pre-GEJE data.

c Secondary medical care areas in Miyagi Prefecture consist of the following four areas: 1) Sen-nan area (Shiroishi City, Kakuda City, Zao Town, Shichikashuku Town, Ogawara Town, Murata Town, Shibata Town, Kawasaki Town, Marumori Town), 2) Sendai area (Aoba Sendai City, Miyagino Sendai City, Wakabayashi Sendai City, Taihaku Sendai City, Izumi Sendai City, Shiogama City, Natori City, Tagajo City, Iwanuma City, Watari Town, Yamamoto Town, Matushima Town, Shichigahama Town, Rifu Town, Taiwa Town, Osato Town, Tomiya Town, Ohira Village), 3) Osaki/Kurihara area (Kurihara City, Osaki City, Shikama Town, Kami Town, Wakuya Town, Misato Town), and 4) Tome/Ishinomaki/Kesen-numa area (Ishinomaki City, Kesen-numa City, Tome City, Higashi-matsushima City, Onagawa Town, Minami-sanriku Town). Municipalities belonging to the secondary medical care area are listed in parentheses.

d The average value between 2009 and 2010 was defined as the suicide rate in this study.

### Statistical analysis

We performed an ecological correlation analysis in each of the 39 municipalities in Miyagi Prefecture. Firstly, we calculated simple correlation coefficients (Spearman’s rho) between the proportion of the population aged 65 years or older, population density of the residential area, unemployment rates, the proportion of all workers engaged in primary industry, suicide rate, the estimated number of inpatients with mental illness, and the prevalence of serious mental distress. Secondly, we categorized the population density of residential areas, unemployment rate, the proportion of all workers engaged in primary industry, suicide rate, and the estimated number of inpatients with mental illness into binary variables (high or low), in order to examine the relationship between the proportion of the population aged 65 years or older and the prevalence of mental distress. If population density in residential areas was below 1,000 it was defined as low; conversely a density of 1,000 or greater was defined as high. If the estimated number of inpatients with mental illness was less than one, it was defined as low; an estimated number of at least one was defined as high. Other variables were categorized based on the median value. Finally, we calculated simple correlation coefficients between the proportion of the population aged 65 years or older and the prevalence of serious mental distress, for each binary variable category. P-values <0.05 were considered statistically significant.

## Results

**Fig 3:**
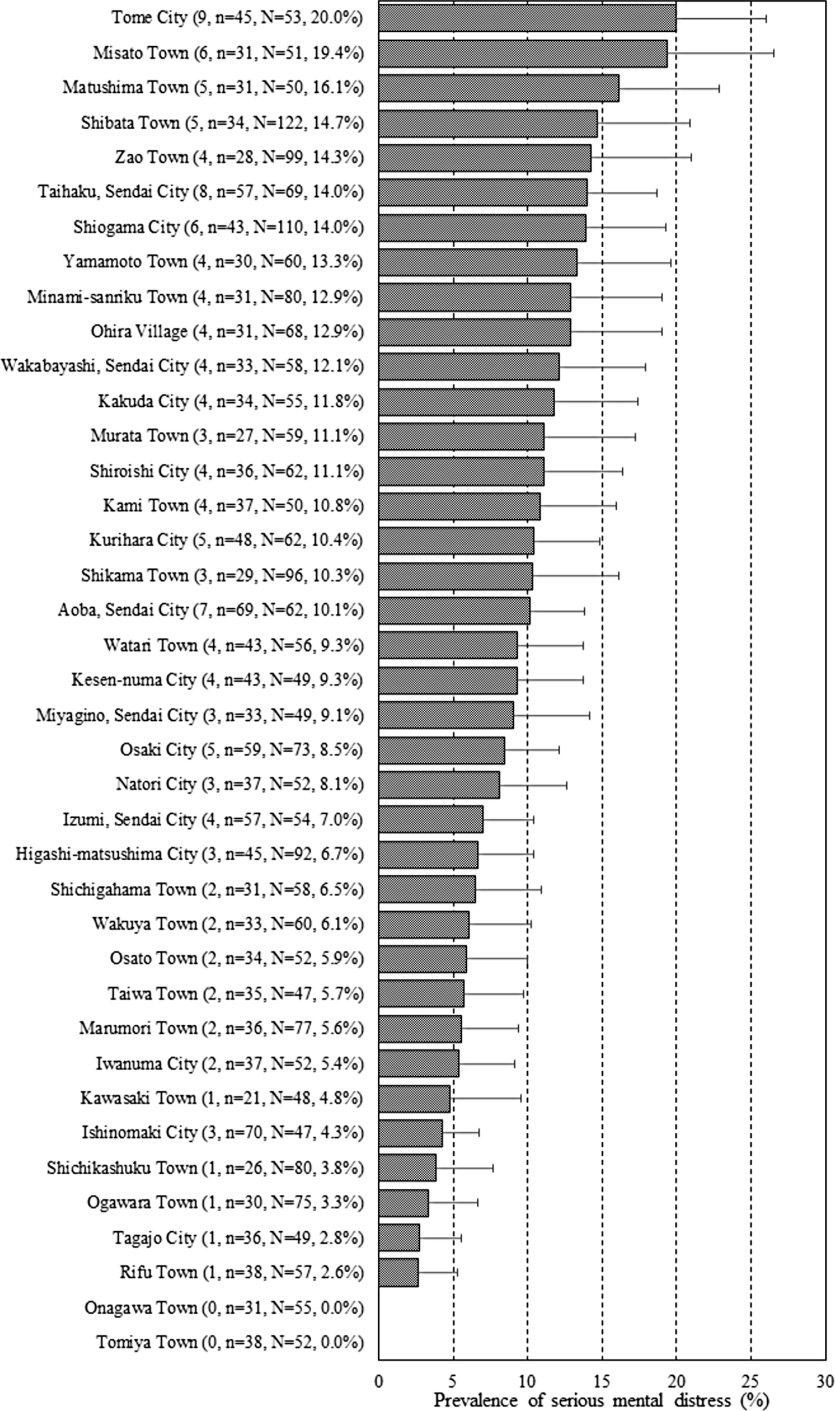
Prevalence of serious mental distress in Miyagi Prefecture. Prevalence of serious mental distress was stratified by municipality (N = 39). Values in parentheses represent those with mental distress, respondents (n), sample size (N), and the prevalence of serious mental distress (%), respectively.

**Fig 4:**
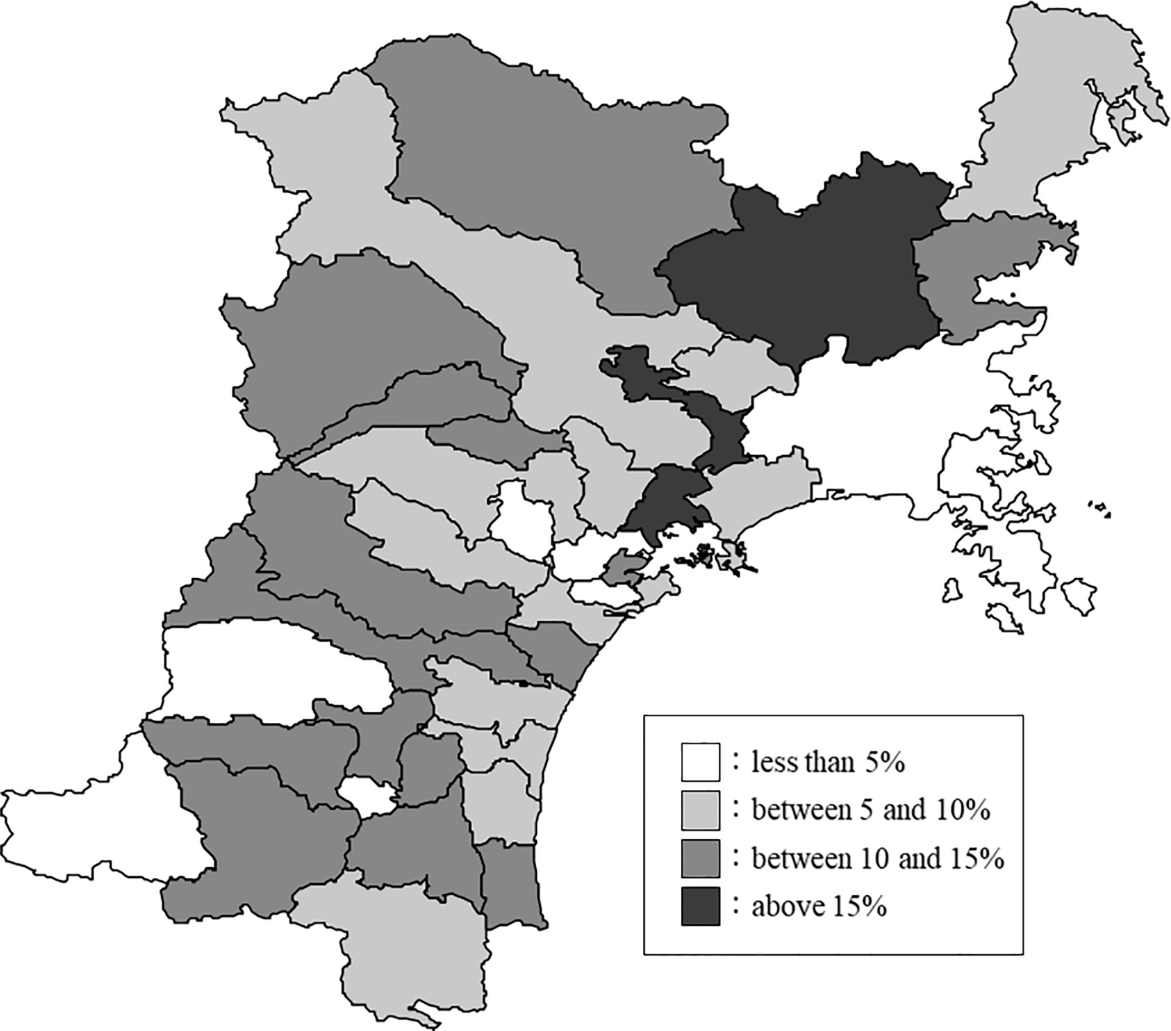
Map showing the prevalence of serious mental distress in Miyagi Prefecture. The prevalence of serious mental distress is color coded every 5%. The figure was edited and processed by the authors using the National Land Numerical Information (Administrative Zones Data) by Policy Bureau, Ministry of Land, Infrastructure, Transport and Tourism.

Of the 1,487 surveyed participants included from Miyagi Prefecture, 9.1% (n = 135) had serious mental distress (Fig 3). The highest and lowest serious mental distress prevalence rates were 20% in Tome City and 0% in Onagawa and Tomiya Towns, respectively. The serious mental distress rate was distributed without bias in the specific areas (Fig 4). Table 3 shows the descriptive statistics for mental distress and social structure in Miyagi Prefecture. The mean proportion of the population aged 65 years or older was 25.4%. The mean population density of the residential area, unemployment rate, the proportion of all workers in primary industry, suicide rate, and the estimated number of inpatients with mental illness were 1067.2, 7.7%, 8.6%, 26.6, and 1.3, respectively.

**Table 3:** Descriptive statistics for mental distress and social structure in Miyagi Prefecture.

		n	(%)	Mean	Median	Standard deviation	Min	Max
Prevalence of serious mental distress		39		9.1	9.3	4.8	0.0	20.0
The proportion of the population aged 65 years or older		39		25.4	27.1	6.2	13.6	44.2
Population density of residential area		39		1067.2	570.6	1119.9	55.2	3718.9
	Low	25	(64.1)	371.9	336.2	217.2	55.2	941.4
	High	14	(35.9)	2308.7	2346.6	994.6	1023.4	3718.9
Unemployment rate		39		7.7	7.9	1.3	4.6	10.0
	Low	19	(48.7)	6.6	6.6	0.9	4.6	7.9
	High	20	(51.3)	8.7	8.6	0.6	7.9	10.0
The proportion of all workers engaged in primary industry		39		8.6	8.8	6.6	0.5	26.8
	Low	19	(48.7)	3.1	2.6	2.4	0.5	8.0
	High	20	(51.3)	13.9	13.6	4.7	8.8	26.8
Suicide rate		39		26.6	27.2	7.2	9.8	44.7
	Low	19	(48.7)	20.8	21.5	4.2	9.8	26.2
	High	20	(51.3)	32.3	31.1	4.3	27.2	44.7
The estimated number of inpatients with mental illness		39		1.3	0.5	1.0	0.2	2.4
	Low	21	(53.8)	0.4	0.5	0.1	0.2	0.5
	High	18	(46.2)	2.4	2.4	0.0	2.4	2.4

We found the prevalence of serious mental distress was not statistically significantly correlated to the proportion of the population aged 65 years or older or any other social structural variables (Table 4). We found a statistically significant correlation between the proportion of the population aged 65 years or older and population density of the residential area (*r*_*s*_ = −0.662, *P* <0.001), the proportion of all workers engaged in primary industry (*r*_*s*_ = 0.798, *P* <0.001), and the estimated number of inpatients with mental illness (*r*_*s*_ = −0.584, *P* <0.001).

**Table 4:** Correlation coefficients for associations between the prevalence of serious mental distress and social structure.

	The prevalence of serious mental distress	The proportion of the population aged 65 years or older	Population density of the residential area	Unemployment rate	The proportion of all workers engaged in primary industry	Suicide rate	The estimated number of inpatients with mental illness
The prevalence of serious mental distress	1.000	0.216	-0.127	0.192	0.096	0.175	-0.178
		(0.186)	(0.442)	(0.240)	(0.559)	(0.287)	(0.277)
The proportion of the population aged 65 years or older		1.000	-0.662	-0.147	0.798	0.182	-0.584
			(<0.001)	(0.371)	(<0.001)	(0.268)	(<0.001)
Population density of the residential area			1.000	0.267	-0.857	-0.344	0.563
				(0.100)	(<0.001)	(0.032)	(<0.001)
Unemployment rate				1.000	-0.343	-0.090	0.381
					(0.032)	(0.587)	(0.017)
The proportion of all workers engaged in primary industry					1.000	0.263	-0.676
						(0.105)	(<0.001)
Suicide rate						1.000	-0.410
							(0.009)
The estimated number of inpatients with mental illness							1.000

Values in parentheses represent P-values

**Fig 5:**
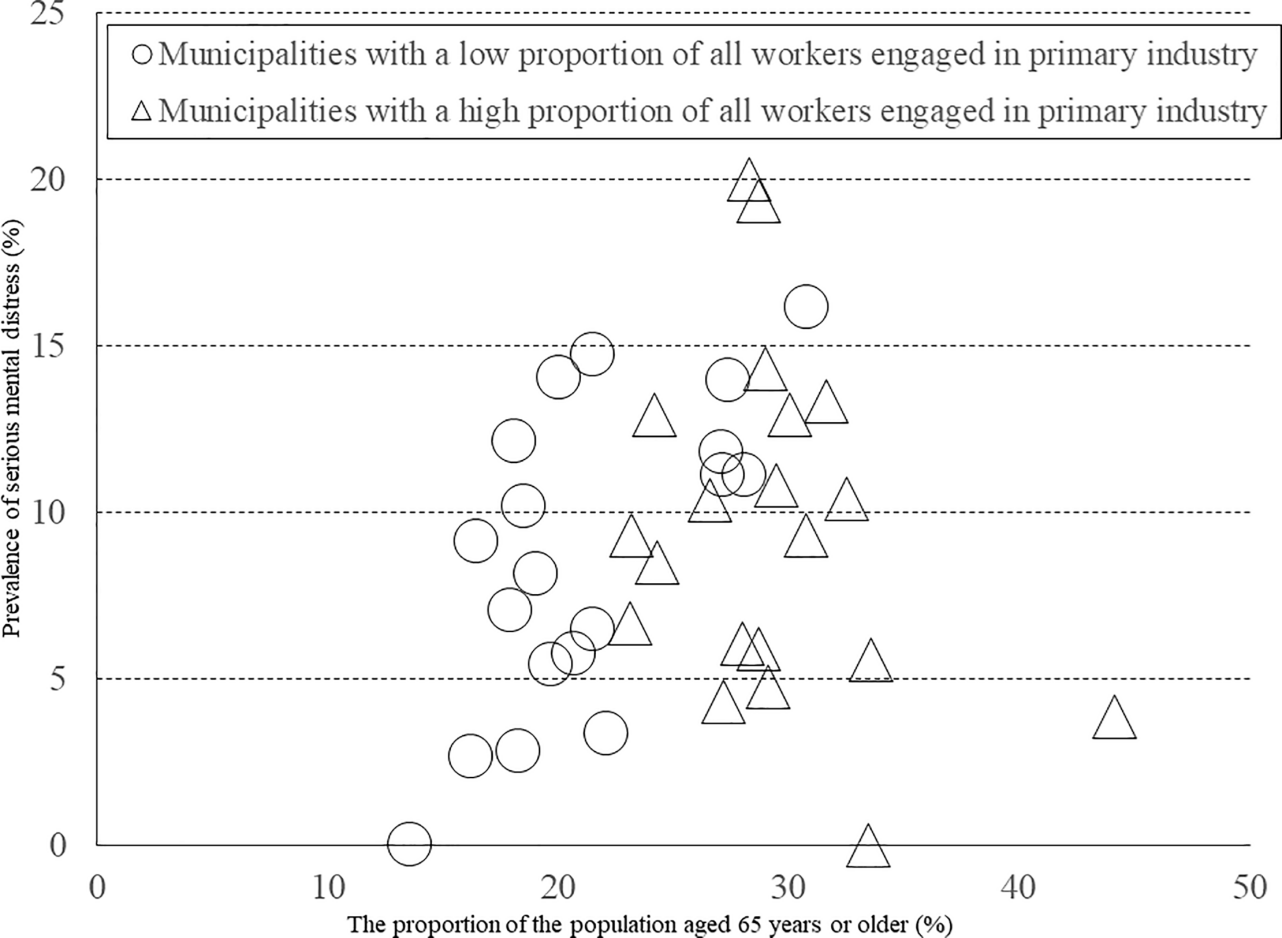
Correlation between the prevalence of serious mental distress and the proportion of the population aged 65 years or older among municipalities by the proportion of all workers engaged in primary industry. The circles represent municipalities with a low proportion of all workers engaged in primary industry (*r* = 0.648, *P* = 0.003); the triangles represent municipalities with a high proportion of all workers engaged in primary industry (*r* = −0.267, *P* = 0.255).

**Fig 6:**
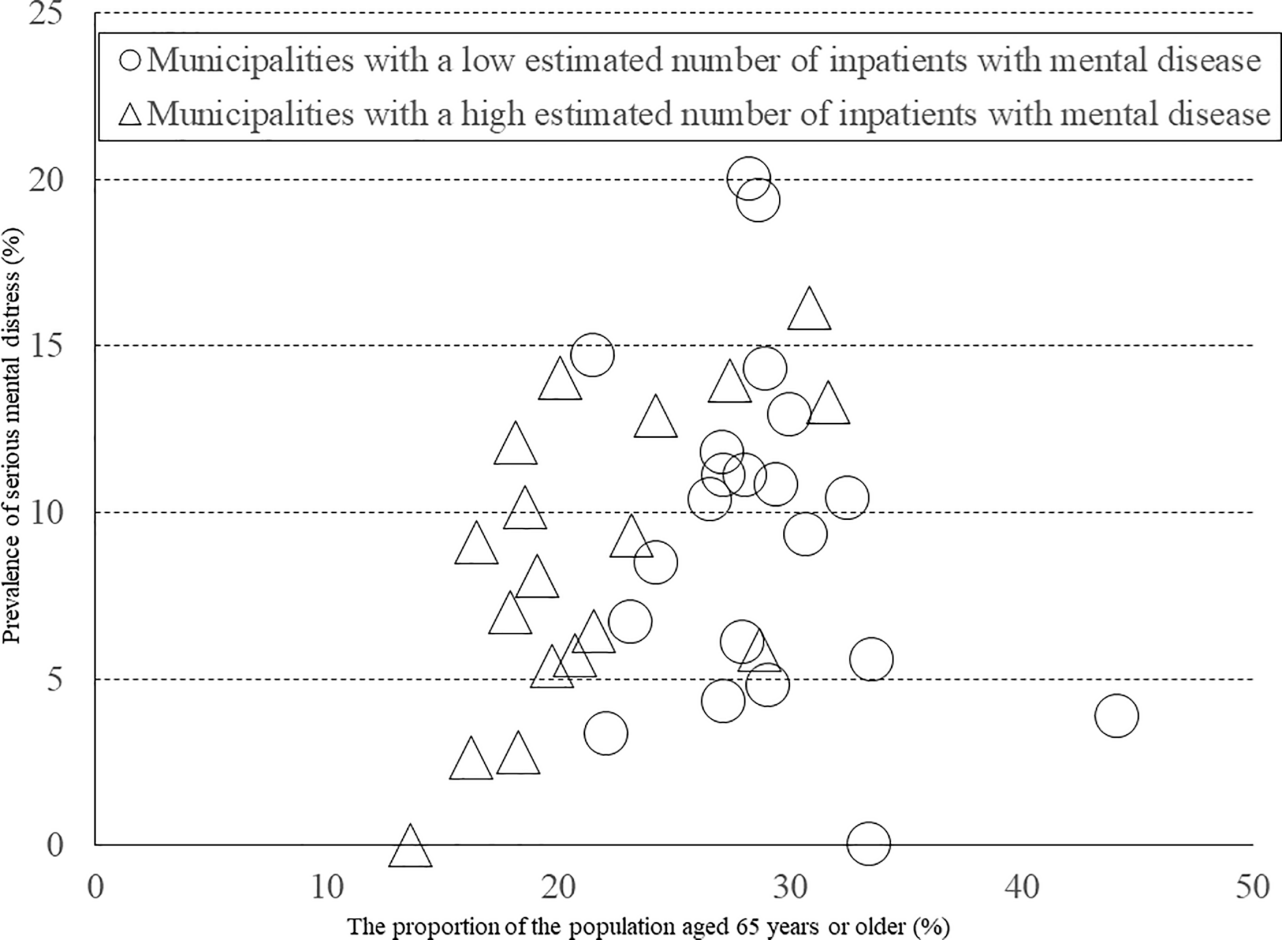
Correlation between the prevalence of serious mental distress and the proportion of the population aged 65 years or older among municipalities by category of the estimated number of inpatients with mental illness. The circles represent municipalities with a low estimated number of inpatients with mental illness (*r* = −0.247, *P* = 0.280); the triangles represent municipalities with a high estimated number of inpatients with mental illness (*r* = 0.626, *P* = 0.005).

Table 5 shows the correlation coefficients between the proportion of the population aged 65 years or older and the prevalence of serious mental distress, for each binary variable category. Even in municipalities with low or high population density, the correlation between the proportion of the population aged 65 years or older and the prevalence of serious mental distress was not statistically significant. Similar results were found for the rate for unemployment and suicide. Regarding the municipalities with a low proportion of all workers engaged in primary industry (Fig 5), we found a statistically significant positive correlation between the proportion of the population aged 65 years or older and the prevalence of serious mental distress (*r* = 0.648, *P* = 0.003). Similarly, we found a statistically significant positive correlation between the proportion of the population aged 65 years or older and the prevalence of serious mental distress in municipalities with a high estimated number of inpatients with mental illness (*r* = 0.626, *P* = 0.005) (Fig 6).

**Table 5:** Correlation coefficients between the proportion of the population aged 65 years or older and the prevalence of serious mental distress, for each binary variable category.

Population density of the residential area		Unemployment rate		The proportion of all workers engaged in primary industry		Suicide rate		The estimated number of inpatients with mental illness	
Low	High	Low	High	Low	High	Low	High	Low	High
0.006	0.016	0.097	0.317	0.648	−0.267	0.323	−0.117	−0.247	0.626
(0.978)	(0.957)	(0.694)	(0.173)	(0.003)	(0.255)	(0.177)	(0.624)	(0.280)	(0.005)

Values in parentheses represent P-values

## Discussion

We determined that the prevalence of serious mental distress before the GEJE in Miyagi Prefecture was 9.1%. Since this study showed the results of a randomly sampled social survey, taking into account the population size of each municipality, this result of the prevalence of serious mental distress would be reasonable. When compared to a study conducted in 2006, that reported that 3% of general Japanese population had serious mental distress [31], it appears that the prevalence of serious mental distress in this study is high. Further, a report on the prevalence of serious mental distress in the general population using a nationwide internet survey, conducted one year after the GEJE [12], also found the prevalence of serious mental distress was 8.9%, which was slightly lower than that in our study. Thus, the prevalence of serious mental distress in Miyagi Prefecture was higher than that of Japan before and after the GEJE. The findings suggest that Miyagi Prefecture may have social structural features that affect mental distress.

Our findings suggest that residents in Miyagi Prefecture could have suffered from poor mental health before the GEJE. Therefore, this may question the accuracy of the findings from previous studies that emphasized that the GEJE affected mental health status of residents. Our study, which accurately describes the distribution of serious mental distress before the GEJE, will be useful for post-disaster studies, providing fundamental data from before the GEJE. However, since the subjects from severely damaged areas had a higher prevalence of serious mental distress than that reported in our study [9,10], the GEJE likely affected mental health among the residents in the highly affected areas.

Further, we found a large difference between the maximum and minimum prevalence rates of serious mental distress based on municipality. Moreover, the prevalence of serious mental distress was distributed without bias in the specific areas investigated. Our findings suggest that mental health could be influenced by social structural characteristics of municipalities themselves, because the prevalence of mental distress was not regionally intensive. Thus, it is important to clarify the relationship between social structure and mental health to ascertain solutions to mental health problems.

Although the proportion of the population aged 65 years or older was not significantly correlated with the prevalence of serious mental distress directly, we found a significant correlation between the prevalence of serious mental distress and the proportion of population aged 65 years or older among municipalities with a low proportion of all workers engaged in primary industry. These results suggest that mental health problems arise among those with difficulties dealing with social structural problems present in an aged society, in the areas where the proportion of all workers engaged in primary industry was low. Generally, it is considered that areas with a low proportion of all workers engaged in primary industry are urban, not rural. However, the correlation between the prevalence of serious mental distress and the proportion of the population aged 65 years or older was not affected by the degree of population density as an urbanization determinant of social structure. Therefore, industrial determinants of social structure, not urbanization determinants, are a crucial influence on the relationship between an aged society and mental health problems. Therefore, in order to solve mental health issues at a social level, it is desirable to manage industrial problems related to an aged society in areas with advanced industrial structure.

On the other hand, the unemployment rate as an economic determinant of social structure was not directly associated with mental distress. Moreover, the degree of unemployment rate did not affect the correlation between the prevalence of serious mental distress and the proportion of the population aged 65 years or older. Our findings differ from those from previous studies, which indicated that unemployment was associated with mental distress [11,20,21]. This is probably because economic problems are greatly affected by an aged society in Japan. Based on our findings, unemployment rate is unlikely to be the main factor, driving mental distress, and an aged society was one of the factors driving mental distress in areas with advanced industrial structure.

We also found a significant correlation between the prevalence of serious mental distress and the proportion of the population aged 65 years or older among municipalities with a high estimated number of inpatients with mental illness. These results suggest that health determinants of social structure are related to the relationship between mental health and an aged society. Thus, because mental health could be affected by physical and mental changes related to aging, it is desirable to put in place measures to mitigate the various physical and mental problems associated with an aged society in areas with a greater number of mental health patients at a social level.

As previously argued, an aged society has an impact on serious mental distress in areas with advanced industrial structure and with a greater number of patients with mental illness. According to Table 4, there was a strong negative correlation between the proportion of all workers engaged in primary industry and the estimated number of inpatients with mental illness. This result suggests that the development of industrialization might be associated with the onset of mental illness. Therefore, we believe it is important to verify the impact of an aged society on serious mental distress, from the viewpoint of the social structure of industrialization and the number of patients with mental illness. Thus, creating a map of the social structural features of the proportion of all workers engaged in primary industry and the estimated number of inpatients with mental illness will be useful for the materials to be used for the survey after the GEJE. We present the colored map of these two social structural features in a supporting information file for the future studies (S1 Fig).

This study was subject to several limitations. We did not take into account demographic characteristics in this ecological study; further studies are needed which should investigate these factors. Furthermore, as the sample size of this survey was not large, care should be taken when interpreting the results. However, since it was scientifically conducted by random sampling, we believe the results are valid and show the distribution of the prevalence of mental distress before the GEJE. Since this was a cross-sectional study of social structure, we did not take longitudinal trends of social structure into account. As the unemployment rate and the proportion of the population aged 65 years or older, in particular, have changed over the last 30 years, it is necessary to examine whether long-term trends are related to mental distress. The K6 evaluated the psychological distress, but it does not directly evaluate mental illness. In the context of population aging in the future, it is necessary to examine the association between K6 and index that could directly evaluate people with psychiatric disorders, such as the elderly population.

We found that residents in Miyagi Prefecture suffered from poor mental health before the GEJE. It is primarily more important to address the social structural problems associated with an aged society in areas with advanced industrial structure and with a greater number of patients with mental illness in order to tackle the mental health issues. Interventions for mental health should not be conducted on a smaller-scale, or targeted at events such as the GEJE, as this will result in turning a blind eye to the present social structural problems of the aged society that are driving mental health problems.

We believe that our findings could greatly contribute to the study of mental distress post-disaster. Following the GEJE, researchers have explored various factors for solving mental problems; however, they have focused on the impact of the GEJE on mental health, disregarding the prevalence of mental distress that might have already been present in the population. We should approach mental health problems in the context of social structure, especially an aged society, taking into consideration facts about mental distress before the GEJE.

## Supporting information

S1 Dataset Mental distress dataset by social survey. (XLSX)

S2 Dataset Social structural dataset. (XLSX)

S1 Fig Map showing the proportion of all workers in primary industry and the estimated number of inpatients with mental illness in Miyagi Prefecture. The figure is color coded by the combination of the proportion of all workers in primary industry (high/low) and the estimated number of inpatients with mental illness (high/low). The figure was edited and processed by the authors using the National Land Numerical Information (Administrative Zones Data) by Policy Bureau, Ministry of Land, Infrastructure, Transport and Tourism.
(TIF)
