# Supplementary figures and images for: The prevalence of mental distress before the Great East Japan Earthquake and the associated impact of an aged society: An ecological study
Source: PLoS One. 2018 Sep 26;13(9):e0203985. doi: 10.1371/journal.pone.0203985 (PMC6157873; doi:10.1371/journal.pone.0203985)

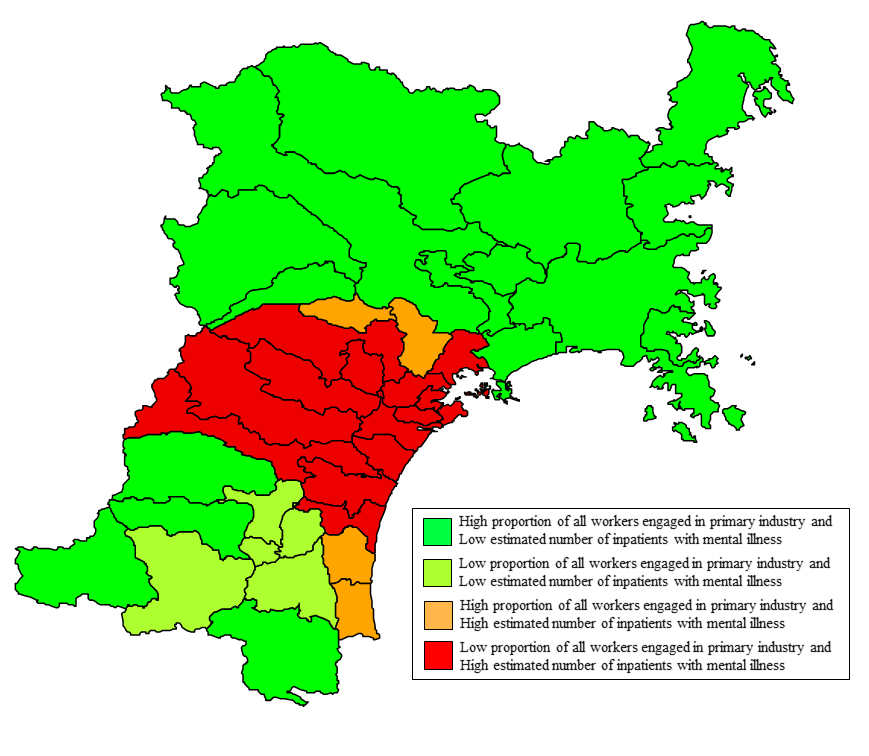

Supplement: S1 Fig — The figure is color coded by the combination of the proportion of all workers in primary industry (high/low) and the estimated number of inpatients with mental illness (high/low). The figure was edited and processed by the authors using the National Land Numerical Information (Administrative Zones Data) by Policy Bureau, Ministry of Land, Infrastructure, Transport and Tourism. (TIF) [file pone.0203985.s003.tif]
